# Effect of reminder phone calls in the language of origin on colorectal cancer screening participation among immigrants in Norway: a randomised controlled trial

**DOI:** 10.1016/j.lanepe.2026.101648

**Published:** 2026-03-13

**Authors:** Nadia Iqbal, Sameer Bhargava, Solveig Hofvind, Khadra Samia Farah, Øyvind Holme, Edoardo Botteri, Paula Berstad

**Affiliations:** a Cancer Registry of Norway, Norwegian Institute of Public Health, Oslo, Norway; b Institute of Health and Society, Faculty of Medicine, University of Oslo, Oslo, Norway; c South-Eastern Norway Regional Health Authority, Oslo, Norway; d Department of Health and Care Sciences, The Arctic University of Norway, Tromsø, Norway; e Stovner District, Oslo, Norway; f Clinical Effectiveness Research Group, Institute of Health and Society, University of Oslo, Oslo, Norway; g Research unit, Sørlandet Sykehus HF, Kristiansand, Norway

**Keywords:** Colorectal cancer screening, Immigrants, Randomised controlled trial, Health equity, Oral information, Language of origin

## Abstract

**Background:**

The effect of providing oral information in immigrants’ language of origin on screening participation remains unclear. This study aimed to assess the effect of a reminder phone call in the language of origin combined with a reminder letter in Norwegian, compared to only a reminder letter in Norwegian, on participation in ColorectalScreen Norway among immigrants from Pakistan and Somalia.

**Methods:**

We conducted a randomised controlled trial among individuals born in Pakistan or Somalia (age 55–56) who did not return a faecal immunochemical test (FIT) within six weeks after the kit was mailed. Individuals were randomised (1:1), to receive a reminder letter in Norwegian and a phone call in their language of origin (intervention) or solely the reminder letter (control). The primary outcome was participation in ColorectalScreen Norway, defined as return of the kit within 12 weeks after sending the reminder letter. ClinicalTrials.gov: NCT06324409.

**Findings:**

We included 416 individuals (215 men and 201 women) during the period from March to October 2024: 217 in the intervention group and 199 in the control group. Overall participation was 24% (53/217) in the intervention group and 11% (22/199) in the control group (risk ratio 2·21 (95% confidence interval (CI) 1·40–3·49)). Participation among Pakistani immigrants was 30% (30/101) in the intervention group and 12% (12/101) in the control group (risk ratio 2·50 (95% CI 1·36–4·60)), and among Somali immigrants 20% (23/116) and 10% (10/98), respectively (risk ratio 1·94 (95% CI 0·97–3·88)) (p_heterogeneity_ = 0·5110).

**Interpretation:**

Reminder phone calls in the individuals’ language of origin doubled the participation rate among immigrants from Pakistan and Somalia in ColorectalScreen Norway, with consistent effects across both nationality groups. The results highlight the complexity and diversity of ensuring access to cancer screening for all and demonstrate the potential of oral information to reach immigrants.

**Funding:**

The Norwegian Cancer Society.

**Research in context**

**Evidence before this study**

We searched PubMed for studies published between January 1, 2010 and March 24, 2025 using combinations of the search terms “cancer screening”, “colorectal cancer screening”, “immigrants”, “migrants”, “oral information”, “reminder calls”, “phone calls”, “native language”, “mother tongue”, “language of origin”, and “randomised controlled trial”. Further, we scanned the reference list of identified papers for relevant studies and also included one study that we came across outside the search. The search was restricted to English and Scandinavian languages. We reviewed all studies on providing oral information about cancer screening in immigrants’ languages of origin, with a particular focus on randomised controlled trials (RCT), especially those conducted in Europe.

Immigrants consistently have lower participation rates in cancer screening compared to non-immigrants across high-income/Western countries. We have previously performed a randomised controlled trial where written translated information to five immigrant groups did not increase participation in breast cancer screening. Previous research suggests that providing oral information in an individual's language of origin may help to increase screening participation among immigrants; however, this approach has not yet been evaluated in a population-based RCT as a primary intervention.

**Added value of this study**

As far as we know, this is the first RCT testing the effect of oral information in immigrant groups, with completed screening participation as the outcome. We found that reminder phone calls in the language of origin can increase participation rate among immigrant groups, specifically among immigrants from Pakistan and Somalia in ColorectalScreen Norway.

**Implications of all the available evidence**

Oral information in immigrants' languages of origin holds great potential in reaching immigrants and increasing participation in cancer screening programs among immigrants. There is still a considerable way to go before implementing such an intervention, as feasibility, including cost-effectiveness, must be considered. The oral information approach should also be refined to maximise its effectiveness in increasing screening participation among immigrants.

## Introduction

Colorectal cancer (CRC) is among the most common cancer types worldwide and in Norway.^1^^,^^2^ CRC is a leading cause of cancer death accounting for 9·3% of cancer deaths globally.^1^ Norway has one of the highest incidences of CRC in the world.^1^ However, the incidence of CRC is lower in many immigrant groups in Norway compared to non-immigrants.^2^^,^^3^ This could change as more immigrants grow older and reach the age where CRC becomes more common. Studies have also shown that both the risk and mortality of CRC among immigrants approach the levels of the host country with increasing length of residence.^4^^,^^5^ Secondary prevention, such as screening, has been shown to reduce CRC incidence and mortality and is recommended by European policymaking institution and international health research agency.^6^^,^^7^

Equitable access to cancer screening remains a challenge. Several studies have shown that cancer screening rates are significantly lower among immigrants compared to non-immigrants.^8^, ^9^, ^10^ In Norway, participation rate for faecal immunochemical test (FIT) was 46% for immigrants compared to 60% for non-immigrants in a pilot prior to the startup of ColorectalScreen Norway in 2022.^8^ Participation rates for immigrants from Pakistan and Somalia were 28% and 18%, respectively. Immigrants from Pakistan and Somalia are populous immigrant groups in Norway, comprising 2·5% and 2·9% of the total immigrant population, respectively, in 2025.^11^

Lower screening rates among immigrant groups can potentially increase their risk for cancer morbidity and mortality. Consequently, there is a demand for interventions capable of increasing screening rates within these communities. In our previous research, we tested an intervention distributing translated invitations for mammographic screening and accompanying written materials to several immigrant groups in the nationwide breast cancer screening program, BreastScreen Norway.^12^ The intervention did not significantly affect the participation rates. Other studies have had similar findings.^13^^,^^14^

Previous research has shown that immigrants, among other things, express a need for verbal information about cancer screening.^15^^,^^16^ To advance knowledge in this field, we conducted a randomised controlled trial (RCT) where the primary aim was to assess the effect of oral information on participation in ColorectalScreen Norway among immigrants from Pakistan and Somalia. Our secondary aim was to evaluate potential variations in the intervention's impact on participation between the two immigrant groups.

## Methods

### Study design

We conducted a two-armed, non-blinded, pragmatic, superiority RCT with a one-to-one allocation, stratified by country of birth (Pakistan and Somalia) within ColorectalScreen Norway. The subgroup analysis by country was pre-specified in the study protocol.

ColorectalScreen Norway, a nationwide screening program, was launched in May 2022. Following a staggered implementation, the program became fully nationwide in December 2023. The program invites all individuals aged 55–65 to participate in CRC screening with FIT every second year. The invitation process involves sending an information letter in Norwegian about CRC screening two weeks before mailing a FIT-sample kit along with a user manual on how to do the test.

FIT is a stool sample taken at home and mailed to a laboratory in a prepaid return envelope. The test detects occult blood in faeces and the threshold for a positive FIT in Norway is 15 μg of haemoglobin per gram faeces. A positive FIT leads to an invitation to colonoscopy at a screening centre located at a nearby hospital. If a stool sample is not received in the laboratory within six weeks after the sample kit was mailed, a reminder letter in Norwegian is sent to the non-participants. Information about CRC screening and the reminder letter are sent digitally to those who have registered a digital account and by post to those without a digital account (<5% in our population). The sample kit with a user manual is sent by post to everyone.

Individuals targeted by the screening program, their gender, country of birth as well as residence time defined as years since registered immigration to Norway were identified through the National Population Register. Most individuals had lived in Norway for over 20 years, so we used a 25-year cut-off for residence time in Table 1 to achieve a better balance between groups. Phone numbers were retrieved from the Contact and Reservation Register.

**Table 1:** Descriptive characteristics of the study population, overall and stratified by immigrant group.

Group	Overall n = 416		Pakistani n = 202		Somali n = 214	
	Intervention n (%)	Control n (%)	Intervention n (%)	Control n (%)	Intervention n (%)	Control n (%)
Total	217	199	101	101	116	98
Gender						
Males	113 (52)	102 (51)	51 (51)	57 (56)	62 (53)	45 (46)
Females	104 (48)	97 (49)	50 (49)	44 (44)	54 (47)	53 (54)
Screening centre						
North and Middle Norway	13 (6)	10 (5)	<5	<5	13 (11)	9 (9)
Southwest Norway	18 (8)	15 (8)	<5	7 (7)	15 (13)	8 (8)
Southeast Norway excluding Oslo/Akershus	41 (19)	32 (16)	11 (11)	11 (11)	30 (26)	21 (21)
Oslo/Akershus	145 (67)	142 (71)	87 (86)	82 (81)	58 (50)	60 (61)
Residence time in Norway						
<25 years	86 (40)	73 (37)	24 (24)	18 (18)	62 (53)	55 (56)
≥25 years	129 (59)	124 (62)	77 (76)	82 (81)	52 (45)	42 (43)
Missing	2 (1)	2 (1)	0	1 (1)	2 (2)	1 (1)

We conducted a pilot of the study during the period from January 2024 to March 2024 by contacting 10 Pakistani and 10 Somali people in the target group of ColorectalScreen Norway in their languages of origin. The pilot study focused on identifying potential practical, administrative and logistical challenges that needed to be addressed to optimise the main study design; therefore, participation in CRC screening was not assessed during the pilot phase. The study period was from March 6, 2024, to January 22, 2025.

### Ethical approval

Ethical approval from the Regional Committees for Medical and Health Research Ethics (REK) in Norway was not required, as the study did not involve direct medical or behavioural interventions. Access to individual-level health data without consent was granted via a dispensation from the duty of confidentiality, as obtaining consent from all participants was not feasible. The study was approved for exemption from the duty of confidentiality under § 29 of the Health Personnel Act and § 19 e and § 20 of the Health Register Act (# H-350) by the Norwegian Health Data Service. The trial is registered at ClinicalTrials.gov: NCT06324409.

### Participants

Our study population consisted of immigrants from Pakistan and Somalia. Various regions of Norway were represented, but about 70% resided in the capital city of Oslo and nearby municipalities as 78% and 50% of all immigrants from Pakistan and Somalia, respectively, resided in the capital area (Oslo/Akershus) in 2025.^17^ We included individuals born in Pakistan and Somalia who received an invitation for CRC screening for the first time and were eligible to receive a reminder in the screening program as they had not returned a stool sample within six weeks after the sample kit was mailed out. We excluded individuals where the reminder letter was returned due to failed delivery (only applicable to those without a digital account) or where the stool sample was recorded as received within three days after the reminder letter was sent. The latter group had most likely sent the stool sample before receiving the reminder letter.

Although the target group for ColorectalScreen Norway is individuals aged 55–65 years, the program was only recently introduced and started by including only one cohort (those who turned 55 in 2022) at the time of its launch and adds one cohort (those turning 55) each year. As a result, individuals included in the study were 55 years old.

### Randomisation and masking

We generated a list of random integers (1 or 2) via random.org and assigned each invitee to a group by matching their sequential internal invitation ID to the corresponding list entry. All invitees were randomized, but we extracted data only for individuals meeting the inclusion criteria; country of birth being Pakistan or Somalia, receiving a first-time invitation to CRC screening and eligible for a reminder.

A data manager identified individuals born in Pakistan and Somalia who did not return a stool sample to the laboratory within six weeks after the sample kit was mailed. Information on group randomisation for the relevant individuals was then retrieved. The identified people were randomised to receive a reminder letter in Norwegian and a phone call in their language of origin (intervention) or solely the usual reminder letter in Norwegian (control).

Due to the nature of the intervention, the callers were aware of the identity of individuals in the intervention arm but were not involved in group allocation. Similarly, blinding individuals in the intervention arm was not possible. None of the individuals in either arm was informed about the trial.

### Procedures

Individuals in the intervention arm received reminder phone calls in Urdu for the Pakistani group and in Somali for the Somali group. During the calls, the purpose of CRC screening was explained to the individuals to ensure informed decision-making regarding participation. Additionally, instructions on how to collect and send a faecal sample were provided. The individuals were also informed about the next steps in the event of a positive or negative test result and were given the opportunity to ask questions. Phone calls were conducted by NI for the Pakistani group and by SKF for the Somali group, both fluent in the individuals’ respective languages of origin and able to switch to Norwegian if desired.

We used a phone call guide to structure our calls (S1). While the phone call information had to be customised for each individual, for instance as they might have asked different questions, we strove to follow a pre-prepared script to ensure uniformity in the information provided to all individuals. The script comprised an initial introduction of the caller and the purpose of the call, followed by requesting permission to provide information regarding CRC screening, and concluded by inviting the individuals to ask questions. A new sample kit could be sent by the caller if requested. The length of the phone call, as well as information on whether the individual ordered a new test kit during or outside the phone call was recorded. In addition, we documented our own experiences with the reminder phone calls throughout the study.

We made two attempts to contact each individual. Following the initial phone call attempt, a standardised SMS in Norwegian was sent to specify that the call was initiated by ColorectalScreen Norway to provide information in the individual's language of origin.

### Outcome

The outcome was a stool sample received in the laboratory within 12 weeks after sending the reminder letter. The 12-week period was chosen because 95% of individuals in ColorectalScreen Norway engage in screening within this timeframe.

### Statistical analysis

We estimated that a sample size of 100 individuals in both the intervention and control groups ensured 90% power to compare two independent proportions, at a two-tailed significance level of 0·05, separately for each nationality group. Based on previous literature,^14^ we assumed a CRC screening participation rate of 5% among individuals receiving a reminder letter and aimed to detect an absolute increase of at least 15 percentage points following the intervention. In order to be able to analyse the intervention effect for each nationality group (Pakistani and Somali) separately as well as detect possible difference in the effect between the groups, we included a total of 200 with Pakistani and 200 with Somali nationality in the trial.

Although not all individuals in the intervention group could be reached, all were retained in the analysis, according to the intention-to-treat principle. Participation was reported as the number of individuals returning the kit divided by those invited. To compare participation rates between the intervention and control groups, we used the chi-square test. A two-sided p-value of <0·05 was considered statistically significant. The primary effect size measure was risk ratio (RR) which was an estimate of the difference in the participation rate between the intervention and control groups. We calculated the RR by dividing the proportion of individuals in the intervention group who returned the FIT by the corresponding proportion in the control group. Ninety-five percent confidence intervals (95% CIs) for the RRs were computed using the Wald method.

To evaluate whether the effect of the intervention differed by country of birth or gender, we fitted multivariable logistic regression models including the study arm (intervention vs. control), the possible effect modifier (Pakistan vs. Somalia or men vs. women), and their interaction, and reported the p-value for the interaction. We also stratified the analysis by years since immigration (<25 y and ≥25 y) and area of residence/screening centre (Oslo/Akershus and rest of the country). In addition, we conducted a sensitivity analysis using Poisson regression with robust standard errors, adjusting for country of birth, gender, years since immigration, and area of residence/screening centre, and reported the result as RR with 95% CI. Missing values for years since immigration (residence time in Norway) were included in the analysis by including a separate category for missing values.

All data were pseudonymized by a data manager, and the statistical analyses were conducted in a secure area on the computer server that required special clearance. We used Stata version 18·5 for Windows for the statistical analyses.

This RCT is reported in accordance with the CONSORT guidelines. Given the minimal-risk nature of the intervention, no formal Data Monitoring Committee or Trial Steering Committee was established.

### Role of the funding source

This study was funded by the Norwegian Cancer Society. The funder of the study had no role in study design, data collection, data analysis, data interpretation, or writing of the report. The Norwegian Institute of Public Health had overall legal responsibility for the conduct of the trial.

## Results

**Fig. 1:**
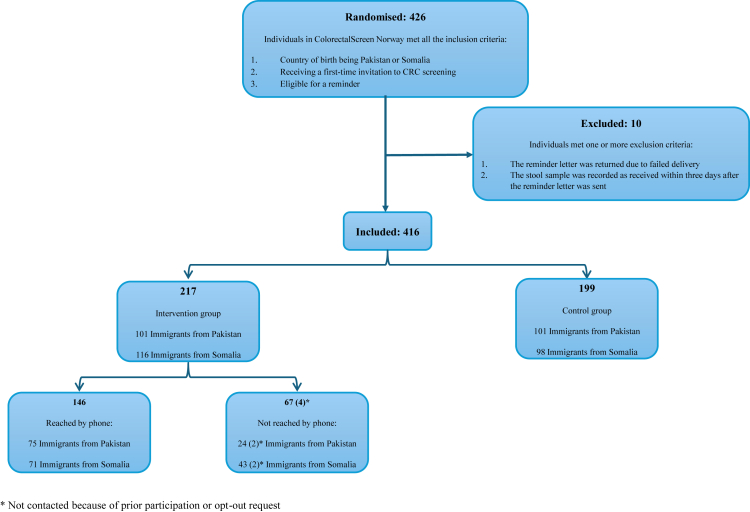
Flow diagram of individual randomisation and inclusion in the intervention and control groups.

A total of 416 individuals were included in the study in the period from March 6, 2024 to October 22, 2024, and follow-up ended January 22, 2025 (Fig. 1). The individuals were randomly distributed between the intervention and control groups in terms of age, gender, place of residence, and length of stay in Norway (Table 1). The intervention group comprised 217 individuals (113 men and 104 women), and the control group comprised 199 individuals (102 men and 97 women). We successfully reached 67% (146/217) of the individuals in the intervention group by telephone.

**Fig. 2:**
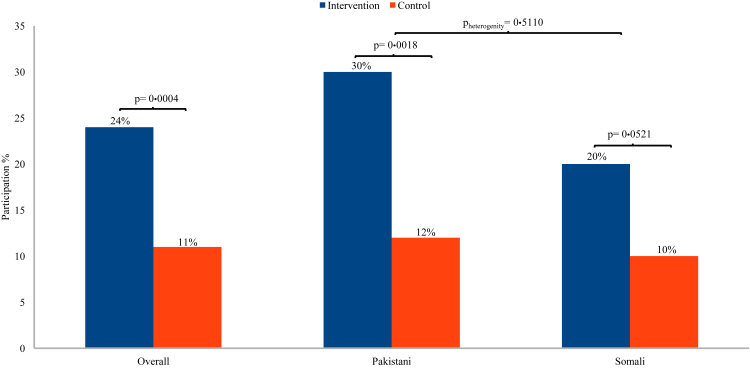
Participation in ColorectalScreen Norway in the reminder phone call intervention group and the control group, overall and by immigrant group.

**Fig. 3:**
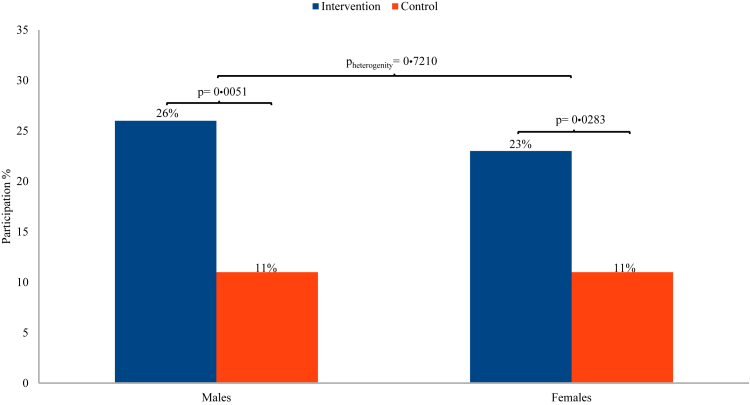
Participation in ColorectalScreen Norway in the reminder phone call intervention group and the control group, by sex.

During the study period, overall participation rate in ColorectalScreen Norway was 24% (53/217) in the intervention group and 11% (22/199) in the control group (RR 2·21, 95% CI 1·40–3·49) (Table 2, Fig. 2). Among men, overall participation rates were 26% (29/113) in the intervention group and 11% (11/102) in the control group (RR 2·38, 95% CI 1·25–4·51) (Fig. 3). For women, overall participation was 23% (24/104) in the intervention group and 11% (11/97) in the control group (RR 2·03, 95% CI 1·05–3·93).

**Table 2:** Participation overall and stratified by immigrant group.

	Overall n = 416			Pakistani n = 202			Somali n = 214		
	Intervention n (%)	Control n (%)	RR (95% CI)	Intervention n (%)	Control n (%)	RR (95% CI)	Intervention n (%)	Control n (%)	RR (95% CI)
Both genders	53/217 (24)	22/199 (11)	2·21 (1·40–3·49)	30/101 (30)	12/101 (12)	2·50 (1·36–4·60)	23/116 (20)	10/98 (10)	1·94 (0·97–3·88)^a^
Males	29/113 (26)	11/102 (11)	2·38 (1·25–4·51)	17/51 (33)	8/57 (14)	2·38 (1·12–5·03)	12/62 (19)	3/45 (7)	2·90 (0·87–9·69)
Females	24/104 (23)	11/97 (11)	2·03 (1·05–3·93)^b^	13/50 (26)	4/44 (9)	2·86 (1·01–8·13)	11/54 (20)	7/53 (13)	1·54 (0·65–3·68)

a P_heterogeneity between Pakistani and Somali_ = 0·5110.

b P_heterogeneity between males and females_ = 0·7210.

Among Pakistani immigrants, participation in colorectal screening was 30% (30/101) in the intervention group and 12% (12/101) in the control group (RR 2·50, 95% CI 1·36–4·60). For men, participation rates were 33% (17/51) in the intervention group and 14% (8/57) in the control group (RR 2·38, 95% CI 1·12–5·03). For women, participation was 26% (13/50) in the intervention group and 9% (4/44) in the control group (RR 2·86, 95% CI 1·01–8·13).

Among Somali immigrants, participation was 20% (23/116) in the intervention group and 10% (10/98) in the control group (RR 1·94, 95% CI 0·97–3·88). For men, participation rates were 19% (12/62) in the intervention group and 7% (3/45) in the control group (RR 2·90, 95% CI 0·87–9·69). For women, participation was 20% (11/54) in the intervention group and 13% (7/53) in the control group (RR 1·54, 95% CI 0·65–3·68).

We found no significant differences in the intervention's effect between immigrants from Pakistan and Somalia (p = 0·5110; men: p = 0·9110, women: p = 0·3630), nor between genders overall (p = 0·7210). Results stratified by years since immigration and area of residence/screening centre did not differ from the main results. The sensitivity analysis, adjusted for the potential confounders available to us—country of birth, gender, years since immigration, and area of residence/screening centre—yielded a result similar to the main analysis (i.e., participation in intervention vs control group, RR 2·23, 95% CI 1·41–3·53).

In the intervention group, the participation rate was 29% (43/146) among those we reached, while 14% (10/71) among those we did not reach (p = 0·0134) (Figure S1). In comparison, 11% participated in screening in the control group. Thirty-nine percent of individuals we reached ordered a new test kit, with 32% doing so during and 7% outside the phone call (Figure S2). Among those who ordered during or outside the phone call, 36% and 40% participated, respectively. By comparison, 83% of individuals who ordered among those not reached ultimately participated.

Most phone calls lasted between 3 and 8·5 min (n = 141, mean = 5·9 (SD = 3·7), median = 5·5 (IQR = 3·0–8·5)). The participation rate was lower among individuals with a phone call duration of less than 2 min compared to those with a duration of 2 min or longer (Figure S3). Participation did not differ between individuals recruited early and those recruited later within the intervention group.

Our experience during the study was that the individuals generally responded very positively to phone calls and appreciated the opportunity to ask questions in their language of origin. However, a small number did not wish to be contacted. During the phone calls, some immigrants from Somalia expressed distrust in the authorities and the healthcare system.

Most of the individuals had opened the digital invitation letter for CRC screening, but a considerable number had not and required an explanation about what CRC screening was. Additionally, even among those who had opened the letter, many still needed clarification due to difficulties understanding its content and screening in general.

## Discussion

In this RCT, we found that reminder phone calls in language of origin significantly increased the participation rate by 2·21 from 11% in the control group to 24% in the intervention group among immigrants from Pakistan and Somalia in ColorectalScreen Norway. The intervention effect may have been somewhat stronger among participants of Pakistani origin than among those of Somali origin; however, the difference between the two groups was not statistically significant. No evidence of effect modification by gender was observed. Ordering a new test kit during or outside the reminder phone call did not guarantee participation in the screening program.

Our hypothesis about the preference for oral information is supported by findings from previous qualitative studies. In a study examining access to diabetes preventive health services, Somali women emphasised the preference of oral information in their language of origin.^18^ Our prior qualitative research indicated that many immigrants from Pakistan expressed a preference for oral rather than written information regarding CRC screening.^15^ This was reaffirmed during our study, as individuals responded positively and often expressed gratitude for the phone call in their language of origin informing them about ColorectalScreen Norway. Notably, 29% of the individuals we reached by phone participated in ColorectalScreen Norway—more than twice the participation rate of those we did not reach and those in the control group. However, the rate was lower than what we anticipated.

Thirty-nine percent of the individuals we reached in the intervention group ordered a new test kit. However, the somewhat smaller increase in participation may be attributed to factors such as the practical steps for completing a stool test being more complex and time-consuming than initially anticipated and the well-documented intention-behaviour gap, where individuals' stated intentions do not consistently translate into corresponding actions.^19^ It is worth noting that only 36% and 40% of those who ordered a new test kit during or outside the phone call, respectively, ultimately participated. Participation was lower among individuals we spoke to for less than 2 min, likely because they did not receive sufficient information about CRC and the screening method. Additionally, some Somali immigrants had low trust in the authorities and healthcare system, which may reflect wider social and cultural challenges. An additional phone call with more information and guidance to complete the test might have facilitated individuals’ completion of the test procedure.

A navigator who supports individuals over time and provides guidance as needed has been shown to increase participation rates among immigrants in breast- and colorectal cancer screening programmes.^20^^,^^21^ Nevertheless, the use of a navigator may raise ethical concerns related to nudging^22^ and patient autonomy and will also be costly and resource demanding for the service provider. Furthermore, one might argue that such a strong nudge could impede the development of individuals' decision-making abilities. However, this type of nudging can foster learning and autonomy by providing essential information and facilitating informed choices.^22^

Studies demonstrating improved participation rates in CRC screening among immigrants have included patient navigation, educational interventions and Afrocentric screening initiatives.^20^^,^^23^^,^^24^ These studies included oral information but did not isolate and examine the effect of such intervention alone. An Australian study demonstrated a large effect (6% vs. 64%) of reminder calls made in the individuals' language of origin.^14^ However, the measured outcome was booking an appointment for mammographic screening, not completing the screening itself. In our study, we observed that a higher number of individuals requested a new test kit compared to the lower proportion of those who ended up participating. The gap between ordering a new test kit and actual participation likely reflected the intention-behaviour gap, where intentions did not always translate into actions and may have had multiple underlying causes. Some individuals may also have ordered a new test kit out of politeness rather than genuine intention.

Several factors have been shown to influence participation in CRC screening among immigrants, including language barriers, knowledge and awareness about CRC and CRC screening, as well as cultural and psychological factors.^25^ Our previous research demonstrated that translation of written material and invitation letter alone was insufficient to increase participation rates in mammography screening.^12^ The challenges affecting participation in cancer screening programmes are diverse and often interconnected, which may necessitate a multifaceted approach to address them effectively. Different immigrant groups can also be in need for different measures to be reached. However, implementing multiple or combined interventions may be resource demanding and are frequently constrained by concerns over cost-effectiveness. This underscores the importance of research focus when developing straightforward, impactful, and resource-efficient strategies to increase screening participation.

At the same time, it is crucial to acknowledge the complexity involved in the decision to participate in cancer screening. While oral communication in the language of origin holds great potential, a single, brief, structured phone call may not suffice to fully inform and educate individuals about cancer screening, or to provide necessary instructions on how to properly complete the test. A lack of knowledge and awareness about cancer and cancer screening has been identified as a barrier to participation and linked to lower screening rates in several studies.^25^, ^26^, ^27^

Furthermore, cancer screening is recommended by the health authorities in Norway, but it is also emphasised that participation is voluntary. One or several phone calls can be perceived as nudging. However, if health service providers aim to implement effective measures to ensure healthy lives and promote well-being for all ages—the third goal of the United Nations' 2030 Agenda for Sustainable Development^28^—it becomes imperative to address inequities in access to healthcare services, including cancer screening. Reducing these inequities is critical for fostering greater inclusivity and equity in public health initiatives.

Participation in cancer screening programmes is closely linked to health literacy, which depends on, among other factors, an individual's ability to gain access to, understand and apply information to maintain and promote good health.^29^ Migrants often score lower on literacy and health literacy measures, necessitating tailored strategies to effectively reach this group.^30^ Integrating oral communication in the language of origin is likely an essential component of such strategies. The overarching goal, however, must always be to empower immigrants to make informed decisions. This raises an important discussion about how we determine if this goal has been achieved, even in cases where participation rates in screening programmes do not increase. Such reflections are vital to ensure that the focus remains on informed autonomy rather than solely on numerical outcomes.

A strength of this study is the inclusion of two large non-Western immigrant groups in Norway. The study is a logical continuation of our previous research showing no effect of the approach with written translated invitations to cancer screening in these groups. Our trial was powered to show the effect of an absolute increase of at least 15 percentage points following the intervention in each nationality group separately. The legal possibility to identify the country of birth of each invited individual in ColorectalScreen Norway from the National Population Register was a necessity which enabled the implementation of the study, which may have limited other institutions to perform similar RCTs.

A potential limitation is that most individuals were from Oslo or neighbouring municipalities. However, this likely mirrors the actual distribution, as both immigrant groups are predominantly concentrated in and around Oslo.

In an earlier study, we found that several factors, such as education, occupation and income, were associated with lower participation among both immigrants and non-immigrants.^8^ These factors were not available for the current study and could therefore not be included in the analyses.

Despite multiple attempts, we were unable to reach all individuals in the intervention group. This limitation could have influenced the results, as outcomes might have differed if all individuals had been contacted and reached. Nonetheless, we believe this reflects a realistic scenario, where not everyone can be reached through phone calls. It also allowed us to demonstrate that the participation among those we did not reach was similar to that of the control group.

The phone calls in each nationality group were conducted by a single individual following a structured guide. While this approach may have introduced the potential for person-dependent bias, it ensured that all individuals within a group received consistent information. Additionally, the conversations were conducted as dialogues, allowing for variations in how the information was conveyed while being tailored to each individual's needs. A small number of individuals (fewer than five) in each group were already acquainted with the caller. It was explicitly stated that the purpose of the phone call was to provide general information about CRC and CRC screening.

Finally, the number of individuals included can also be considered a limitation. Although the sample size was determined a priori, the observed effect of the intervention was lower than anticipated, which may have reduced the statistical power of the study, especially for subgroup analyses. This should be considered when interpreting the results.

The results of this study highlight the complexity of ensuring accessibility to cancer screening for all. However, the results also demonstrate the potential of incorporating oral information into strategies aimed at reaching immigrants. The intervention, while effective, is unlikely to be cost-effective in its current form. Further refinement is needed before potential implementation. Our study offers valuable insights for healthcare providers, authorities, policymakers, organisations focused on immigrant health, and those involved in preventive healthcare.

We conclude that reminder phone calls in language of origin significantly increased participation in ColorectalScreen Norway among immigrants from Pakistan and Somalia. The effect did not differ by nationality or gender, and ordering a new test kit was not necessarily followed by participation. Future interventions should consider incorporation of oral information in the individuals’ language of origin to reduce barriers related to low health literacy to promote equitable access to healthcare services for immigrants.

## Contributors

PB, SB, and SH designed the study. PB, SB, SH, and NI conducted the literature review. NI, EB, and PB have directly accessed and verified the underlying data reported in the manuscript and did the statistical analyses. NI is responsible for drafting and editing the manuscript with inputs from SB and PB. All authors contributed to the discussion, critically reviewed the manuscript, and approved the final version of the submitted manuscript. The corresponding author affirms that all listed authors meet the authorship criteria and that no one meeting the criteria has been omitted.

## Data sharing statement

Research data used in the analyses can be made available upon request, provided there is a legal basis under GDPR Articles 6 and 9, that processing complies with Article 5, and that any additional national legal requirements for population-based health surveys are met.

## Declaration of generative AI and AI-assisted technologies in the manuscript preparation process

During the preparation of this work the authors used ChatGPT in order to improve language clarity and readability in parts of the manuscript. After using this tool/service, the authors reviewed and edited the content as needed and take full responsibility for the content of the published article.

## Declaration of interests

SB received personal fees from Gilead outside the submitted work. We declare no other competing interests.

## Appendix A Supplementary data

Supplementary FigureS1

Supplementary FigureS2

Supplementary FigureS3
