# Supplementary material for: Effect of reminder phone calls in the language of origin on colorectal cancer screening participation among immigrants in Norway: a randomised controlled trial
Source: Lancet Reg Health Eur. 2026 Mar 13;63:101648. doi: 10.1016/j.lanepe.2026.101648 (PMC13000538; doi:10.1016/j.lanepe.2026.101648)

Figure S2: Participation in ColorectalScreen Norway by ordering a new test kit in the reminder phone call intervention group and the control group

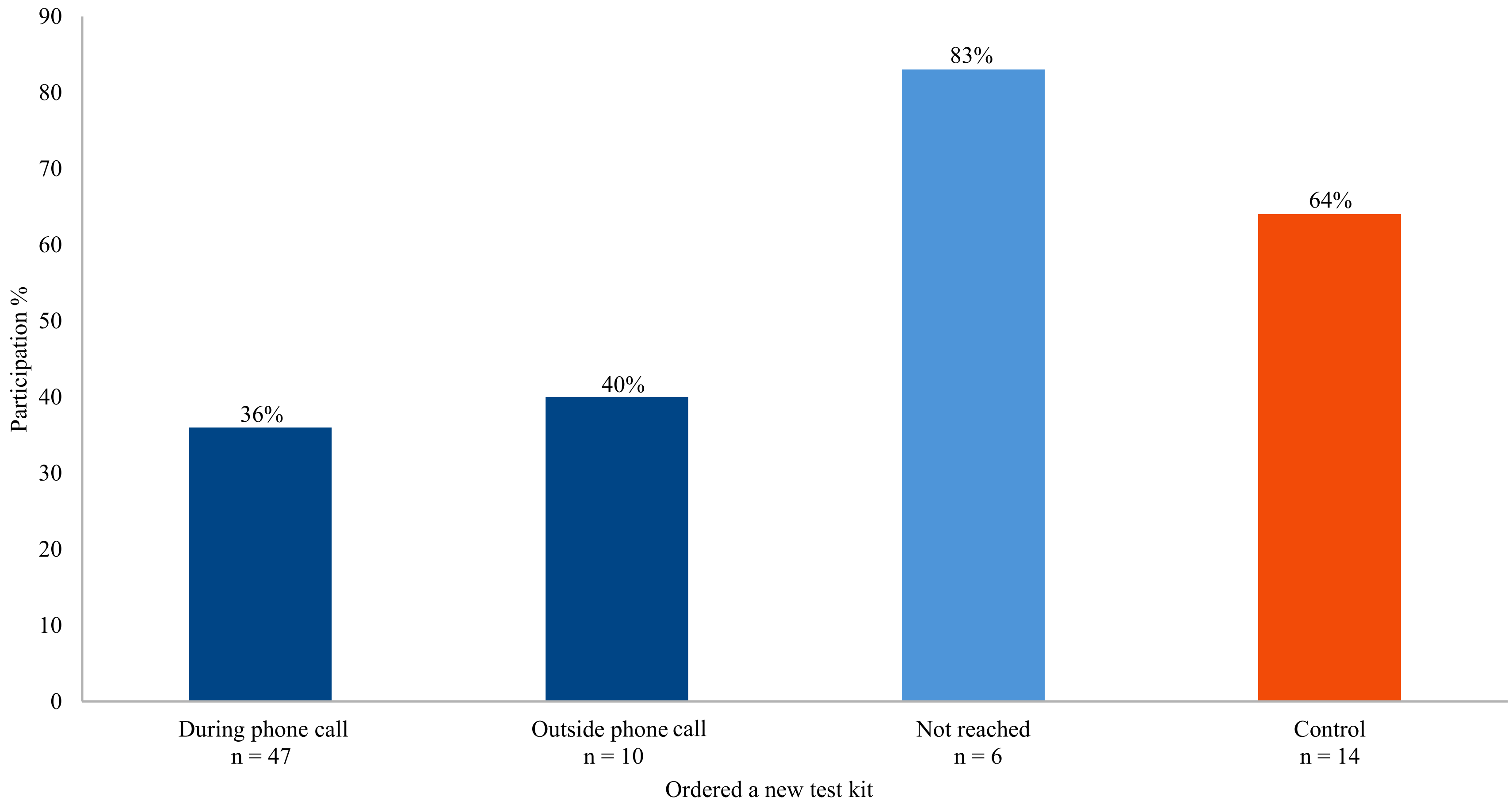

Supplement: Supplementary FigureS2 [file mmc2.pdf]
