# Supplementary material for: Effect of reminder phone calls in the language of origin on colorectal cancer screening participation among immigrants in Norway: a randomised controlled trial
Source: Lancet Reg Health Eur. 2026 Mar 13;63:101648. doi: 10.1016/j.lanepe.2026.101648 (PMC13000538; doi:10.1016/j.lanepe.2026.101648)

Figure S3: Participation in ColorectalScreen Norway by phone call duration in the reminder phone call intervention group and the control group

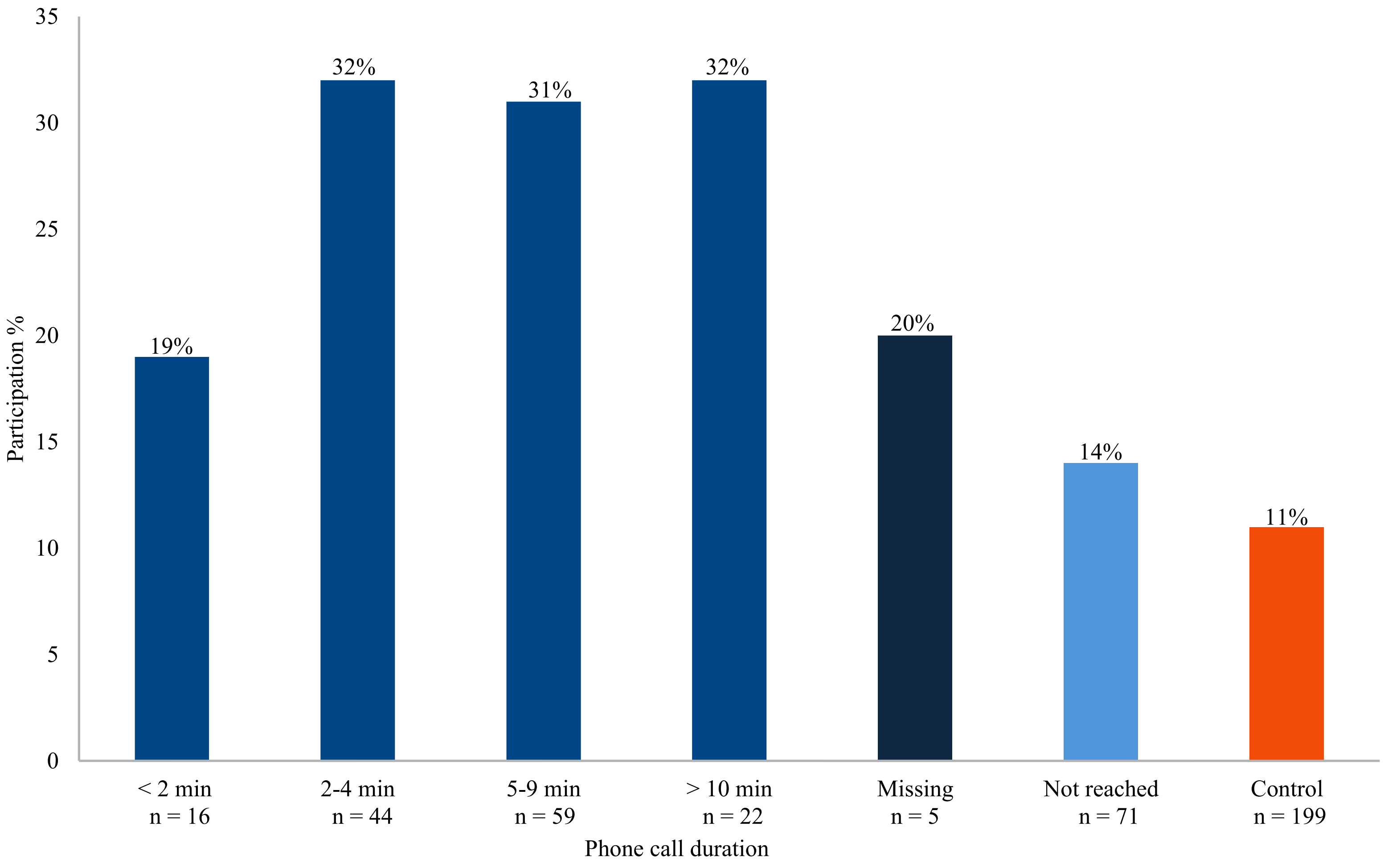

Supplement: Supplementary FigureS3 [file mmc3.pdf]
